# Prediction accuracy of L- and M-cone based human pupil light models

**DOI:** 10.1038/s41598-020-67593-3

**Published:** 2020-07-03

**Authors:** Babak Zandi, Julian Klabes, Tran Quoc Khanh

**Affiliations:** Department of Electrical Engineering and Information Technology, Laboratory of Lighting Technology, Technical University of Darmstadt, 64289 Darmstadt, Germany

**Keywords:** Human behaviour, Visual system

## Abstract

Multi-channel LED luminaires offer a powerful tool to vary retinal receptor signals while keeping visual parameters such as color or brightness perception constant. This technology could provide new fields of application in indoor lighting since the spectrum can be enhanced individually to the users’ favor or task. One possible application would be to optimize a light spectrum by using the pupil diameter as a parameter to increase the visual acuity. A spectral- and time-dependent pupil model is the key requirement for this aim. We benchmarked in our work selected L- and M-cone based pupil models to find the estimation error in predicting the pupil diameter for chromatic and polychromatic spectra at 100 cd/m^2^. We report an increased estimation error up to 1.21 mm for 450 nm at 60–300 s exposure time. At short exposure times, the pupil diameter was approximately independent of the used spectrum, allowing to use the luminance for a pupil model. Polychromatic spectra along the Planckian locus showed at 60–300 s exposure time, a prediction error within a tolerance range of ± 0.5 mm. The time dependency seems to be more essential than the spectral dependency when using polychromatic spectra.

## Introduction

The pupil aperture is an essential factor in photometric and visual investigations because of its direct influence on both retinal illumination and the retinal image quality^1^. A smaller pupil diameter can ensure a larger depth of field^2^ and achieve a decrease of optical aberrations^3,4^, which has positive effects on the visual acuity of the eye^4,5^. Visual acuity is relevant in the interior lighting of workplaces or production facilities since an enhanced visual performance leads to fewer accidents or human injuries^6^. Various studies have shown that the optimal pupil diameter is approximately between two and three millimeters for visual tasks in the photopic luminance range^1,4,7–11^. With today’s technology of multi-channel LED luminaires, it is possible to optimize artificial light spectra to influence the pupil aperture, color perception, brightness perception or other lighting metrics^12,13^. The number of narrow-band light-emitting diodes in such a system determines the degree of freedom, which allows keeping specific parameters constant while changing others. The first step to actively optimize the pupil aperture through illumination without influencing other image-forming vision parameters such as brightness or color perception is the construction of an accurate model which predicts the spectral and time-dependent pupil diameter. Such a model can be used in a heuristic or gradient-based optimization procedure as an objective or constraint function to design the desired light spectrum for visual tasks.

Eight empirical models are proposed in the literature with different dependent parameters and test conditions. The most famous models are from Holladay^14^, Crawford^15^, Moon and Spencer^16^, De Groot and Gebhard^17^, Stanley and Davies^18^, Barten^19^ and Blackie and Howland^20^. In 2012, Watson and Yellot^21^ reviewed these pupil models in a pioneer work and developed a combined pupil model that integrates the effect of luminance, adapting field size, age of subjects and number of eyes into a single unified formula. As a base, the function of Stanley and Davies^18^ was used, which already managed the relationship of pupil diameter as a function of luminance and adaptation field size. The age correction was performed by using the data from Winn et al., who conducted age-dependency experiments^22^. All mentioned pupil models are based on the sensitivity of L- and M-cones, which makes them unsuitable in a light spectrum optimization process to achieve an ideal pupil aperture. Using these models would make it impossible to change the pupil diameter while simultaneously maintaining brightness and color perception. However, an extension or revision of these models would be conceivable for such an application. Before this step can be performed with broader pupil examinations, the question arises how well existing models predict the aperture size at all. Moreover, the missing time-dependence of these models leads to the next question of how important such a parameter could be inside a function. In reality, the pupil diameter shows inter- and intrapersonal scatter. Recent studies have shown that the intrapersonal variance of an individual observer can be approximately up to ± 0.3 to 0.6 mm^23,24^. The interpersonal variation is much higher and can reach up to ± 1.5 mm^15,22^. Cognitive effects which influence the pupil aperture are negligible because they are transient, meaning for a short time of period^21^ with a maximum amplitude of approximately ± 0.4 mm^25^. Therefore, an ideal pupil model could never be more precise than the intrapersonal variance of a single observer. Besides intra- and interpersonal scatter, a significant inaccuracy arises because of the mentioned non-time-dependent nature of existing models and the usage of a photopic luminous efficiency function called V(λ). Integrating a V(λ)-weighted quantity in a pupil model can lead to deviations, especially when using multichannel LED luminaires since existing pupil models were mainly developed with white light from thermal radiators. Recent works conclude that the peak wavelength sensitivity of the pupil does not correspond to V(λ) alone^26–29^ and is highly time-dependent^30^, which results in a peak sensitivity shift during exposure time from 510 nm to approximately 470 nm^30,31^. Thus, the underlying assumption of existing models that the pupil light response is controlled via an achromatic channel consisting of an additive combination of L and M-cones is obsolete. This is mainly due to the discovery of melanopsin expressing intrinsically photosensitive ganglion cells (ipRGCs)^32–37^ in the inner retina which contributed fundamentally to the understanding of visual processing^34,38–42^, form vision^43^, brightness perception^44–50^, circadian photoentrainment^37,51–53^ and pupil light response^54–57^. There are six different subtypes^58,59^ of ipRGCs projecting to the olivary pretectal nucleus^36,41^ (OPN), the dorsal lateral geniculate nucleus^43,60,61^ (LGN) and the suprachiasmatic nucleus^42,62^ (SCN) of the hypothalamus. The so-called M1 ipRGC-subtypes are part of the primary afferent pupillary pathway which controls the pupil aperture through the OPN and the Edinger–Westphal nucleus^63,64^. According to recent investigations, they receive and integrate extrinsic synaptic input from rods, with additive contribution from L- and M-cones, separate opponent inhibitory signals of S-cones^34,65–67^ and M-cones^68,69^. Furthermore, there are indications for an impact of the parvocellular pathway with chromatic red-green signals^69,70^, possibly a separate and independent post-receptoral mechanism^55,70,71^. A 3D reconstruction of the S-cone circuit to the ipRGCs revealed that a novel S-cone amacrine cell has targeted inhibitory synapses to the ipRGCs^72^. Thus, the higher sensitivity of the pupillary light reflex for short wavelengths could also be partly caused by S-cones^72^. The integration mechanism of the classical photoreceptor signals inside the ipRGCs is linear in a vector summation fashion^70^, but with different time-dependent weighting contributions to driving the pupil response under constant light. The proportion varies with stimulus intensity, spectral distribution and change over exposure time. Classical outer photoreceptors mediate the transient pupil constriction^5^ whereby an intrinsic melanopsin activation of ipRGCs^28,73,74^ mainly drives the sustained photopic adapted pupil response. All receptor signals contribute complementary^75^ to the afferent pupil constriction control path with varied proportions until a steady-state of equilibrium is reached, in which the melanopsin activated ipRGC signals are dominating. According to Mure et al., a stabilized state is attained within approximately 3 min of exposure time^28^. Moreover, recent studies show that in mesopic and scotopic adaption the rods are involved in the phasic and sustained pupil light response^27,57,63,75,76^. At higher retinal illumination levels the melanopsin activated ipRGCs are dominating this contribution^2,74^.The time until the pupil diameter has reached its' steady state depends on the used spectrum composition^73,77–79^. McDougal and Gamlin^30^ showed, by fitting a custom receptor weighting function, that cones contribute little to the pupil light response after 30 s. The derived function for determining the receptor contribution did not consider the pupil light reflex's opponent channels. Therefore, it is still unknown how the inhibitory and excitatory synaptic connection from the outer photoreceptors to the ipRGCs are timed to drive the temporal pupil light response.

Based on the discussed findings on the pupil control path, it must be assumed that when using L- and M-cone models, deviations in prediction of the diameter must be expected. The pupil models were developed using empirical data from experiments with white light from thermal radiators. Thus, chromatic LED spectra or monochromatic light stimuli could cause the highest prediction errors. However, the use of white light generated by multichannel LED luminaire can also lead to increased deviations, as LED-systems have a higher degree of freedom in the composition of the spectral distributions. For instance, a chromaticity coordinate on the Planckian locus can be reached with different spectral distributions. Depending on which polychromatic spectrum is used, different receptor weightings can be triggered in the pupil control path. In a previous study^80^, pupil data from monochromatic experiments by Bouma^26^ were used to determine error deviations of the Watson and Yellot model. It was found that by transforming the dependent variable into a melanopsin-weighted radiant flux, only variation for longer wavelengths occurs. This study indicated that the prediction error of an L- and M-based model is mainly due to the lack of a ipRGC-weighting^80^, when it comes to the steady state pupil diameter. Bouma's studies^26^ in 1962 were groundbreaking, but the data are based on the research with one subject, which does not allow conclusions about the scattering in the data. Furthermore, it is unknown whether the stimuli were presented randomly and which adaptation time was used in the conducted experiment by Bouma^26^. The result confirms the finding that the wavelength-dependent sensitivity function of the pupil shifts to the direction of the ipRGC sensitivity with increasing adaptation time. However, the equilibrium state is not always reached at the same time and can vary depending on the used spectrum.

L- and M-cone based pupil models will continue to be important in future, as they allow us to calculate the pupil diameter without knowing the spectrum of a light source. Meanwhile, the luminance of an object can be easily determined by using simple measurement equipment. New research results on the pupil control pathway raise the question of how high the deviation can be when using classical pupil models. This knowledge can also be used to assess the extent to which the classical pupil models are still suitable for a correct time- and spectrally dependent pupil function. We have used a custom build accurate temperature-controlled multi-channel LED luminaire to investigate how high the prediction errors in L- and M-cone based models can be and to what extent this is influenced by the temporal and spectral dependence of the pupil control path. We collected pupil data from an inter- and intrapersonal study to ensure a general statement and to guarantee repeatability of the results. Both, chromatic and polychromatic LED spectra along the Planckian curve as light stimuli with constant luminance were used. The polychromatic spectra were optimized with 15-LED-channels to find out how high the error deviation can be when the spectral distribution of the white light deviates from thermal radiators. With our results, we want to offer the opportunity to other research groups and indoor light designers to select the right model for pupil prediction calculations. We want to reveal the potential benefit of a spectrally time-dependent pupil model to realize the idea of optimized visual light spectra in indoor lighting with a contribution of the pupil aperture as a parameter.

## Methods

### Background and formulas of L- and M-cone based pupil light models

The first known model to predict the pupil diameter was published by Holladay^14^ in 1929, and was based on three subjects of unknown age (Eq. 1). The investigations were conducted with two frosted light bulbs in a homogeneously illuminated chamber. Subjects were adapted 10–15 min to different intensities, and the right pupil diameter was measured manually through a double-pinhole pupillometer. He used an exponential function to model the data, which has the disadvantage of having strong errors at high luminance^21^. Crawford criticized the double-pinhole methodology and described it as undesirable because subjects were not under the sole influence of the light stimulus during pupil measurements^15^. Crawford^15^ built his model in 1936 based on pupil measurements using photographs with a reference object in the image (Eq. 2). He examined ten subjects of unknown age in his study. Light stimulus was achieved by combining a projector lamp with a 55° sized white screen. The Crawford-Model used a hyperbolic tangent function for the first time, which saturates the pupil diameter at high and minimal luminance. He compared his raw data with the study of Reeves^81^, and the results of Holladay. All three authors investigated the sustained pupil diameter with different adaptation times. Crawford used about 5 min in his investigation, Reeves 15 min and Holladay between 10 and 15 min. Crawford found a large variation of approximately one to two millimeters between the data sets, which he said could only be explained by the individual differences of subjects. Moon and Spencer^16^ created a combined model (Eq. 3) in 1944 based on data from five different authors to achieve greater generality. These include the data sets of Blanchard^82^ 1918 (two subjects, 5 min. adaptation), Reeves^81^ 1918 (six subjects, 15 min. adaptation), Crawford^15^ 1936 (eight subjects, 5 min. adaptation), Stiles 1929 (one subject) and Covreux 1924 (one subject). From Crawford's data, eight of ten subjects were included. The adaptation time from Reeves and Covreux is unknown. Thus, the model of Moon and Spencer is based on a total of data sets from 18 subjects with unknown age. Like the Crawford model, the Moon and Spencer model used a hyperbolic tangent function as the basis for fitting data, but the parameters were adjusted so that the maximum possible pupil diameter was higher, and the minimum pupil diameter lower compared to Crawford’s model. The model by De Groot and Gebhard^17^ from 1952 included eleven more subjects (Eq. 4), in addition to the data from Moon and Spencer. The authors used an exponential function like the Holladay model because they criticized that they cannot imagine why the pupil would reach physiologically such a strong saturation state at high luminance, as it would be the case with a hyperbolic tangent function. For the first time, the model of Stanley and Davies^18^ considered not only the photometric intensity but also the adaptation field size of the stimulus, since they assumed that the high variance of data between earlier authors was because of the partly unknown size of the stimulus surface (Eq. 5). The investigations were performed with varying luminance and different adaptation fields between 0.4° and 25.4°. The adaptation fields were circular and the exposure time was 60 s. They constructed their model based on nine subjects of unknown age. Blackie and Howland^20^ developed another pupil model (Eq. 7), using data from Flamant^83^. The intensity range of the available data goes up to 10 cd/m^2^ and is valid for the mesopic adapted pupil diameter. However, the conditions under which the pupil data were measured were not mentioned. The Barten^84^ model from 2009 used Le Grand's^85^ formula from 1968 as a basis and extended this with Bouma's^86^ measurements to include the dependence of pupil diameter on adaptation field size (Eq. 6). Barten did not mention the conditions under which the data from Le Grand were generated. In 2012, Watson and Yellot^21^ summarized the above described seven formulas and transformed the dependent photometric quantity into the derived SI-unit cd/m^2^ (Eq. 1–7). They developed a so-called unified pupil formula, which incorporates the parameters “luminance”, “number of eyes”, “size of the adapting field” and “age” of the subjects (Eqs. 8, 9). As a basis, the model of Stanley and Davies from 1995 was used, who already integrated the size of adaptation field. The correction for the age dependence was approximated from the data set of Winn et al., who conducted experiments with 91 subjects in age from 17 to 83 at five luminance levels between 9 and 4,400 cd/m^2^. The adaptation time in the work of Winn et al. was one minute, and the mean value of the last ten seconds was used as pupil diameter. The dependence of the monocular effect of one or two eyes was integrated with the data sets from Blanchard^82^, Reeves^81^ and ten Doesschate and Alpern^87^. For this, Watson and Yellot added a factor to the basic model of Stanley and Davis.

1 $$ {\text{Holladay 1926}}:D_{H} \left( L \right) = 7 {\exp}\left[ { - 0.1007 L^{0.4} } \right] $$

2 $$ {\text{Crawford 1936}}:D_{C} \left( L \right) = 5 - 2.2 {\tanh}\left[ {0.61151 + 0.447 \log_{10} L} \right] $$

3 $$ {\text{Moon }}\& {\text{ Spencer 1944}}:D_{MS} \left( L \right) = 4.9 - 3 {\tanh}\left[ {0.4\log_{10} L} \right] $$

4 $$ {\text{De Groot }}\& {\text{ Gebhard 1952}}:D_{DG} \left( L \right) = 7.175 {\exp}\left[ { - 0.00092 \left( {7.597 + \log_{10} L} \right)^{3} } \right] $$

5 $$ {\text{Stanley }}\& {\text{ Davies 1995}}:D_{SD} \left( {L,\alpha } \right) = 7.75 - 5.75 \left( {\frac{{\left( {L \cdot \alpha /846} \right)^{0.41} }}{{\left( {L \cdot \alpha /846} \right)^{0.41} + 2}}} \right) $$

6 $$ {\text{Barten 1999}}:D_{B} \left( {L,\alpha } \right) = 5 - 3 {\tanh}\left[ {0.4 \log_{10} \frac{L \cdot \alpha }{{40^{2} }}} \right] $$

7 $$ {\text{Blackie }}\& {\text{ Howland 1999}}:D_{BH} \left( L \right) = 5.697 - 0.658 \log_{10} L + 0.07\left( {\log_{10} L} \right)^{2} $$

8 $$ {\text{Watson }}\& {\text{ Yellot 2}}0{12}:D_{WY} \left( {L,\alpha ,y,e } \right) = D_{SDW} + \left( {y - y_{0} } \right)\left[ {0.02132 - 0.009562 D_{SDW} } \right] $$

9 $$ D_{SDW} \left( {L,\alpha ,e } \right) = 7.75 - 5.75 \left( {\frac{{\left( {L \cdot \alpha \cdot e/846} \right)^{0.41} }}{{\left( {L \cdot \alpha \cdot e/846} \right)^{0.41} + 2}}} \right) $$

According to Barten^84^, the angle in his model should be calculated for a rectangle adapting field with $\alpha = {\alpha }_{x}{a}_{y}$ and for circular fields $\alpha =\pi /4 {D}^{2}$, with D as field diameter in degrees. In the model of Stanley and Davies^18^ and Watson and Yellot, the angle is given with the unit deg^2^. In the Watson and Yellot model, y_0_ stands for the reference age 28.58 years, *e *= 0.1 for one exposed eye and *e *= 1 for two. An implemented toolbox is available in Mathematica and Matlab to calculate the pupil diameter with one of these models. In our work we used the Matlab implementation from Wheatley and Spitschan. When using one of these toolboxes, care should be taken due to approximation formula ${\alpha }_{{deg}^{2}}=\left({\alpha }_{deg}/2\right)\pi$, which converts the adaption field size angle $\alpha$ in degree to the square degree angle in deg^2^. This approximation leads to conversion errors for larger angles. For angles higher than 15°, we recommend the formula ${\alpha }_{{deg}^{2}}=6566\pi \left(1-\mathrm{cos}\left({\alpha }_{deg}\pi /360\right)\right)$. To test the prediction accuracy of pupil models, we used the Crawford model, because it is based on his own experiments, which has the advantage of lower raw data variance. Compared to Holladay's model, we considerd the Crawford model to be more accurate, as the measurement technique from Holladay can lead to higher deviations. The model by De Groot and Gebhard is used as a representative of the combined models for the steady-state sustained pupil diameter. The models from Barten and Blackie and Howland were excluded, because of unknown experimental parameters. We also evaluated the latest pupil formula by Watson and Yellot, which is the only model that combines the parameters “age”, “number of eyes” and “adaptation field size”. Thus, it is unnecessary to evaluate the Stanley and Davies model, since the Watson and Yellot model is based on it. We expect that the model of Crawford and De Groot and Gebhardt should in principle perform better for sustained pupil diameter with polychromatic spectra, since they obtained the data from experiments with longer adaptation times and white light. In contrast, the Watson and Yellot model should perform better for shorter adaptation times. Our hypothesis is that all three models should have higher prediction errors when using chromatic spectra, especially in the short wavelength range, since these models do not take into account the time dependent ipRGCs contribution or any other chromatic channel of the pupil constriction path. Errors can also occur when using white light spectra, since our polychromatic stimuli are mixed with different LED channels and the spectral distribution is significantly different from the spectrum of a thermal radiator.

### Participants

Twenty observers were recruited from the Technical University of Darmstadt to attend two experimental sessions. In the first session we conducted a pupil measurement with chromatic spectra and in the second with polychromatic spectra. Both sessions were carried out separately. One individual subject was tested in-depth with twelve repetitions in each test condition. The prerequisites for participation were an age between 19 and 25 years, no history of ocular disease, no use of medications or drugs that could influence the pupil response, no caffeine and alcohol 48 h before the experiment took place. The subjects in the polychromatic session were aged between 19 and 25 years, mean age 21.95 SD ± 1.73 years. In the chromatic session, the observers were 19–25 years old, mean age 22.2 SD ± 1.77 years. The subject, which took part in the more extensive measurements with twelve repetitions, was 33 years old and is one author of this manuscript. The ethic committee of the Technical University of Darmstadt approved the study (ID: EK 12/2019). Thus, the study was carried out in accordance with the ethical principles of the Declecartion of Helsinki. We met all relevant guidelines and regulations of TU Darmstadt’s ethic comittee. All observers provided a signed consent prior to the experiment and informed consent was obtained from all participants.

### Photometric setup conditions and pupillometry protocol

**Figure 1:**
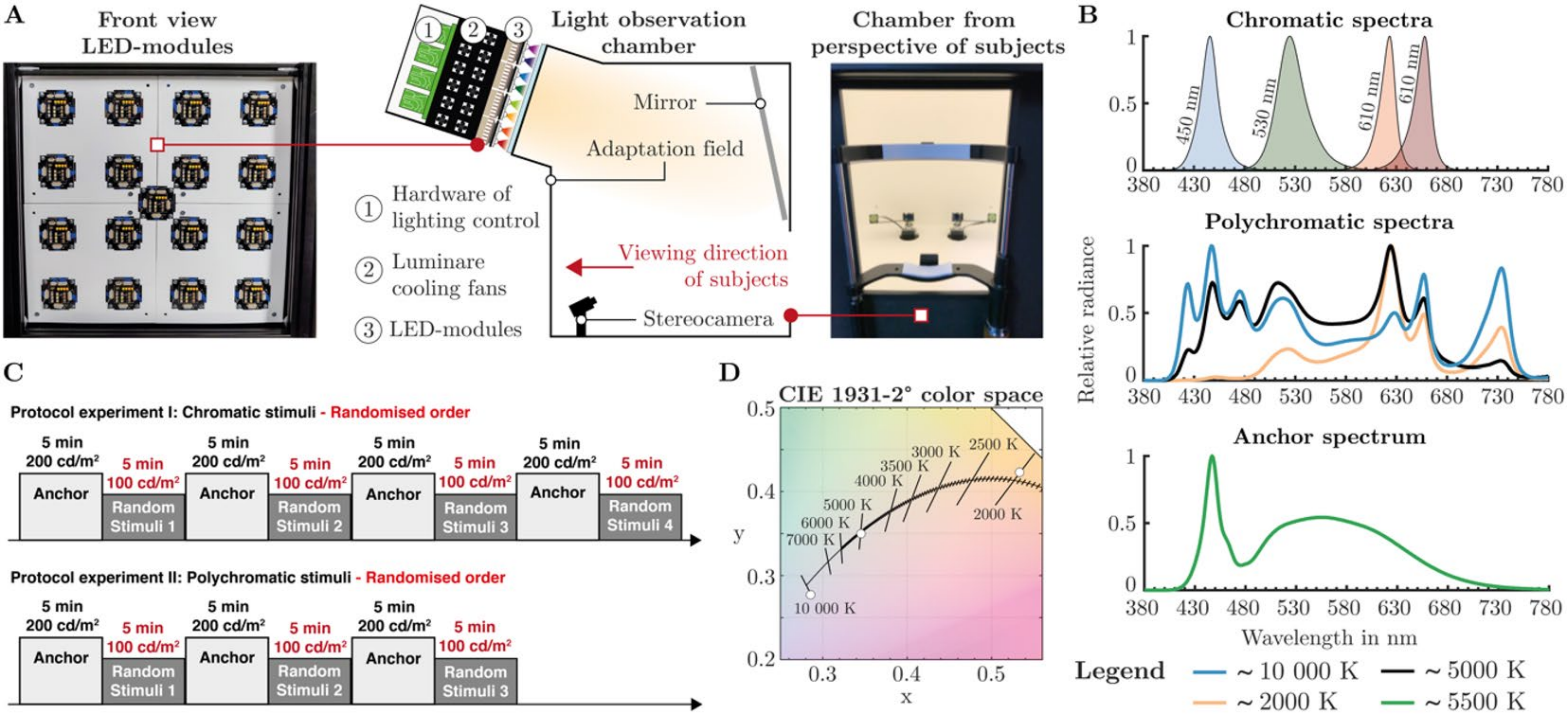
Experimental setup conditions and used stimuli. (**A**) Middle: side view of the observation chamber with custom built temperature controlled 15-Channel-LED Luminaire as light source, positioned on top of the setup. Left: front view of 4 × 4 arranged LED-modules behind luminaire’s diffusor optic. The additional LED-module in the center of the 4 × 4 grid was not used. Each module was cooled to a constant temperature with Peltier elements. Right: illuminated chamber from the observer’s perspective. A chin rest minimized head movements of the observers. Pupil measurements were performed with a stereo camera system. (**B**) Measured Spectra of used stimuli in the chromatic and polychromatic experimental sessions. The anchor spectrum was used as adaptation spectrum. (**C**) Protocol of two performed chromatic and polychromatic experiments in this study. The spectra were presented randomly for 5 min with a constant luminance of ~ 100 cd/m^2^. Before each stimulus spectrum, an anchor spectrum L =  ~ 200 cd/m^2^ was switched 5 min on to adapt observer’s pupil diameter back to a baseline. (**D**) The polychromatic spectra ~ 2000 K, ~ 5000 K and 10 000 K from the second experiment were optimized to lie on the Planckian locus. Chromaticity points are shown in the CIE 1931–2° color space.

We developed a temperature-controlled 15-channel LED-luminaire consisting of eleven narrow-band and four phosphor-converted white LEDs. Peak Wavelengths of the eleven narrow-band light-emitting diodes were 420 nm, 450 nm, 470 nm, 505 nm, 530 nm, 545 nm, 590 nm, 610 nm, 630 nm, 660 nm, 720 nm and full widths at half maximum were 14 nm, 18 nm, 25 nm, 29 nm, 33 nm, 105 nm, 78 nm, 17 nm, 16 nm, 17 nm, 29 nm. The correlated color temperature of the phosphor-converted white light-emitting diodes were 2700 K, 4000 K, 5000 K and 5500 K. We placed fifteen LEDs with different peak wavelengths on a custom made 50 × 50 mm one-layer aluminum circuit board. We mounted behind each LED-board a fan-cooled heat sink with one Peltier element. Temperature measurement of LED-boards was conducted with two PT100 sensors. One was soldered to the front of the circuit board and another one was glued with temperature conductive adhesive behind the heat sink. The size of the illuminated surface from the multi-channel LED-luminaire was 400 × 400 mm, due to the arrangement of sixteen of this temperature-controlled LED-modules in a 4 × 4 matrix. Empty spaces between the LED modules were lined with 3D-printed white Polylactide elements to avoid light reflections back into the housing (Fig. 1A-left).

Luminance output of each LED-channel was controlled separately with a STM32-Nucleo-F767ZI by pulse width modulation (PWM) through a linear constant-current sink LED regulator. We avoided flicker perception effects by setting the PWM-frequency to 2 kHz. The complete system was housed in a non-transparent black case with optical diffuser glass, mounted on the front of the 4 × 4 LED-module matrix panel. We attached the entire luminaire system on top of an observation box with sufficient space to mix the rays from the light source inside the observation chamber (Fig. 1A-middle). Homogeneous illumination of a 700 × 700 mm rectangular adaptation field was reached using a mirror inside the chamber (Fig. 1A-middle). Both, walls and the bottom floor of the chamber were painted white with custom mixed barium sulfate color to ensure diffuse reflection. During the main experimental time participant’s head position were kept still by a chin rest, and the gaze was held constant through a 0.8° fixation target^88^ in the middle of the adaption field (Fig. 1A-right). These procedures led to a steady viewing angle with minimized pupil foreshortening error^89^ from pupil measurement. Viewing distance to the adaption field was 700 mm, corresponding to a visual angle of 53.1°. We used a fixation target shape from Thaler et al., consisting of a bull-eye and cross-hair combination, which aims to reduce gaze dispersion and micro-saccade rate^88^. The LED-circuit boards were regulated to a temperature of 30 °C ± 0.1 with a proportional-integral-derivative (PID) controller. Thus, our LEDs operated stable and reproducible, without a significant shift of the light spectrum, caused by temperature fluctuations of the light-emitting diodes. The hardware of the lighting system was interfaced with a personal computer through an UART communication protocol. For this, a custom serial communication software was programmed in MATLAB, which offered the possibility to adjust the intensity of each LED-channel via duty cycle. Serial command inputs from MATLAB to a microcontroller naturally have latency times which could cause inaccuracies in the synchronization of pupil data with the switch-on time of the light stimulus. Therefore, we measured the delay time from sending a command in MATLAB to the embedded processing in the microcontroller. The time delay was taken into account when performing the synchronization between the pupil raw data and the light-on time.

Our study was divided into two experiments. In the first experiment chromatic spectra were used with peak wavelengths of 450 nm, 530 nm, 610 nm and 660 nm to determine the maximum deviation of the mentioned pupil models. Since chromatic spectra are an extreme case and do not occur in everyday life, we used polychromatic white spectra in a second experiment with ~ 10 000 K, ~ 5000 K and ~ 2000 K along the Planckian locus. The first step in obtaining specific spectra from a multi-channel LED luminaire is to calculate the duty cycle for each channel from given visual metrics. Classically, gradient based^90^, heuristic optimization^91^ or analytical methods^92^ can be used for this purpose. Due to the high number of LED channels in our setup, gradient-based procedures often stuck in local minima. Therefore, we used a heuristic multi-objective optimization method (genetic algorithm) to calculate the duty cycle of each channel. We specified as main objective values, the CIE 1976 2° u′v′-chromaticity coordinates and the luminance in cd/m^2^. Luminance optimization target was set to 100 cd/m^2^, because we found in pre-studies with chromatic spectra that a transition between two spectra are more pleasant for the observers at such an intensity level. Comparability between the experiments was achieved by keeping the 100 cd/m^2^ in the second experiment with polychromatic spectra. The optimization results were adjusted in the lamp and measured twenty times with a Konica Minolta CS2000 spectroradiometer on each study day. We were able to achieve a mean correlated color temperature of 10 138 SD ± 22 K, 4,983 SD ± 3 K and 2007 SD ± 1 K for the polychromatic spectra (Fig. 1B,D) with an average luminance of 99.8 SD ± 0.2 cd/m^2^. For simplicity, polychromatic spectra in this paper are labeled ~ 10 000 K, ~ 5000 K and ~ 2000 K. The chromatic spectra with peak wavelengths of 450 nm, 530 nm, 610 nm and 660 nm had an average luminance of 100 SD ± 0.2 cd/m^2^ and were manually adjusted without using an optimization procedure (Fig. 1B). Averaged calculated visual metrics of twenty repeated measurements on every experimental day are listed in Table 1. Measured absolute spectra are reported in the supplementary materials (Table S1). The first experiment was conducted with four chromatic stimuli presented in random order, each with an exposure time of 300 s (Fig. 1C). A reference stimuli of 5500 K (Fig. 1B) with 199.45 SD ± 0.43 cd/m^2^ was offered 300 s as an anchor between every chromatic stimuli to avoid pre-stimulation influences^28,30^. One test session took 40 min in total. During this time, observers fixed the target inside the observation chamber. Head movements were minimized using a chin rest (Fig. 1A-right). The same protocol was used in the second experiment with polychromatic spectra. Again, the spectra were presented in random order with an anchor spectrum between the stimuli (Fig. 1C). Stimulus presentation was performed using a custom programmed MATLAB software. The two experiments were conducted independently of each other on different experimental days. A test-leader checked the gaze position of the subjects during every session with real-time gaze tracking. The stimulus spectra were presented at constant luminance because, according to the L- and M-cone based pupil models, the diameter should remain constant over the different spectra.

**Table 1:** Calculated metrics of used spectra in the polychromatic and chromatic experiments.

Stimulus label	Luminance (cd/m^2^)	u′ 1976	v′ 1976	S-Cone	M-Cone	L-Cone	Melanopsin	CCT in Kelvin
Polychromatic 10 000 K	99.83 ± 0.2	0.1985 ± 2.3 × 10^–5^	0.4333 ± 1.1 × 10^–5^	0.1198 ± 2.3 × 10^–4^	0.1524 ± 3.3 × 10^–4^	0.1652 ± 3.7 × 10^–4^	0.1668 ± 3.3 × 10^–4^	10,138 ± 22
Polychromatic 5000 K	100.10 ± 0.2	0.2123 ± 3.4 × 10^–5^	0.4838 ± 6.4 × 10^–5^	0.0636 ± 1.2 × 10^–4^	0.1396 ± 2.8 × 10^–4^	0.1645 ± 3.5 × 10^–4^	0.1225 ± 2.4 × 10^–4^	4,983 ± 3
Polychromatic 2000 K	100.17 ± 0.3	0.3042 ± 3.8 × 10^–5^	0.5430 ± 1.5 × 10^–5^	0.0064 ± 2.1 × 10^–5^	0.1026 ± 2.6 × 10^–4^	0.1709 ± 4.1 × 10^–4^	0.0456 ± 1.2 × 10^–4^	2007 ± 1
LED-Peak 450 nm	99.73 ± 0.4	0.2163 ± 3.3 × 10^–5^	0.0617 ± 1.5 × 10^–4^	3.2458 ± 6.2 × 10^–3^	0.4834 ± 1.2 × 10^–3^	0.2913 ± 8.7 × 10^–4^	1.8173 ± 3.9 × 10^–3^	–
LED-Peak 530 nm	100.12 ± 0.2	0.0649 ± 4.8 × 10^–5^	0.5787 ± 1.2 × 10^–5^	0.0040 ± 1.4 × 10^–5^	0.1672 ± 3.8 × 10^–4^	0.1409 ± 3.3 × 10^–4^	0.1055 ± 2.3 × 10^–4^	–
LED-Peak 610 nm	100.16 ± 0.2	0.4951 ± 7.5 × 10^–5^	0.5256 ± 3.9 × 10^–5^	0.0001 ± 3.5 × 10^–5^	0.0471 ± 1.0 × 10^–4^	0.1968 ± 4.6 × 10^–4^	0.0008 ± 2.8 × 10^–5^	–
LED-Peak 660 nm	99.97 ± 0.2	0.5823 ± 3.0 × 10^–4^	0.5115 ± 1.8 × 10^–4^	0.0008 ± 1.1 × 10^–4^	0.0236 ± 7.1 × 10^–5^	0.2023 ± 4.4 × 10^–4^	0.0011 ± 1.3 × 10^–4^	–

Twenty measurements were taken on every experimental day using a Konica Minolta CS-2000 spectroradiometer. Mean values with standard deviations are listed. Excitation of S-cones, M-cones, L-cones and ipRGCs were computed with the 10-deg cone fundamentals and the melanopic action spectra reported in CIE S 026/E:2018. The Cone and ipRGCs exaltation values are specified as $\alpha$-opic radiance in W/m^2^ sr.

### Pupil measurement and steps of pre-processing

We recorded pupil light responses at 120 frames/s on a multi-camera system from Smart Eye Pro with two 659 × 494 pixels Basler acA640-120gm cameras and 8 mm lenses. Extrinsic and intrinsic camera-calibration was performed with a checkerboard, resulting in an average accuracy of 0.15 mm for edge detection. The gaze calibration was done before each experiment. Camera control, calibration and pupil detection were carried out with the Smart Eye Pro software, which returned the timestamp, pupil diameter in mm, recognized eye blinks and ellipse fitting accuracy of the pupil in percent. During pupil measurements, artifacts usually occur in the raw pupil data, caused by eye blinks, head movements or rapid gaze jumps. Therefore, the pupil data needs to be pre-processed. We removed artefacts caused by eye blinks from the dataset with the blink detection algorithm from the Smart eye pro software. Other non-physiological pupil changes were detected and removed using the stated pupil measure accuracy by the Smart Eye Pro system. Pupil data which had an accuracy of less than 97% were deleted from the dataset. Remaining peaks were cleaned using a velocity filter. For this purpose, the pupil data were numerically differentiated to get the velocity profile from which we removed all strong outliers with a percentile threshold criterion of 99.993% and 0.007%. Missing data were interpolated linearly. Data smoothing was performed with a Savitzky–Golay-Filter over a window size of 3,000 data points. The first three seconds of the data set were not smoothed out, as this would lead to a artificially induced minimization of the short-term pupil diameter. In this work, we only considered the pupil data of the left eye. The evaluation of the results is based on two approaches. First, we performed a significance analysis to find out how much the pupil diameter is affected by the spectrum at constant luminance. The substractive baseline-corrected^93^ pupil diameter from the respective anchor-spectra is used for this (Fig. S2). Second, the pupil models were evaluated. For this, a baseline-correction is not performed, because we compare the deviation of the absolute pupil diameter with the baseline-free estimated prediction of the pupil models. Pupil offset errors^93^ during the measurement should not have a significant effect on our data due to the use of camera calibration, fixed gaze point by using the target and the limited head movements of observers effected by the chin rest.

## Results

Pupil data from both experiments were extracted at exposure times of 1 s, 60 s, 300 s and were compared with the predicted values from chosen pupil models. The estimation error is shown by calculating the difference between the predicted value of the models from Crawford, De Groot and Gebhard, Watson and Yellot and the actual measured pupil diameter of the subjects. Pupil data extraction at 60 s was chosen because the Watson and Yellot model is based on data with an adaptation time of 1 min. The models by Crawford and De Groot and Gebhard used data from sustained pupil diameter; hence we checked the estimation error at 300 s exposure time. We also assessed the prediction error of the short-time pupil diameter to see how well they work in a range where theoretically a dominance of the classical photoreceptors is present. To calculate the predicted pupil diameter, we applied the average luminance 100 cd/m^2^ for the chromatic session and 99.8 cd/m^2^ for the polychromatic trial inside the models. The Watson and Yellot model requires the additional parameters: number of eyes, size of the adaptation area and age of observers. In our experiment, the number of exposed eyes was two, and the visual angle of the adaptation area corresponded to 53.1°. As age parameter we used the mean value of our sample, which was 22.2 years in the chromatic experiment and 21.95 years in the polychromatic trial. For the individual subject, which was tested in detail with 12 repetitions, an age of 33 years was used.

These parameters yield in the chromatic experiment a predicted diameter of 3.007 mm by Crawford, 3.182 mm by De Groot and Gebhard and 3.019 mm, according to Watson and Yellot. In the polychromatic experiment, the changed mean age and the slightly lower luminance results in pupil diameter of 3.006 mm by Crawford, 3.182 mm by De Groot and Gebhard and 3.022 mm using the Watson and Yellot model. The predicted pupil diameter of the individual observer does not change according to the models of Crawford and De Groot and Gebhard, as there is no age dependency in these models. In the Watson and Yellot model, the individual subject’s pupil diameter is 2.942 mm in the chromatic session and 2.943 mm in the polychromatic part. The differences between the models are relatively small. In our conditions, the parameters age and adaptation size had only a tiny influence on the pupil diameter. We expect that in real measurements, the interpersonal scatter would over-shade the importance of these parameters. Based on the results of the used models, one would expect that the pupil diameter would have to remain constant in all used spectra. We assume that the models are more accurate and more independent of wavelength for the phasic pupil response since a combination of the classical photoreceptors controls the pupil. Several studies have shown that the proportion of S-cones is rather weak and that in interpersonal studies, the scattering could mask the effect^66,94^. This would allow an approximately correct assumption by the pupil models, since an estimated description by the L- and M-cones would indeed be possible, due to the masked S-cone influence. However, this would mean that a model has to be able to distinguish between two different time state responses of the pupil. One state for the classical photoreceptor input and another for the sustained pupil response with enhanced ipRGCs proportion. In the next two sections, the results of the chromatic and polychromatic experiment are explained in detail. For this purpose, we firstly performed a statistic on the baseline-corrected pupil diameter, to check the conditions under which the luminance could be useable as a quantity inside a pupil model. Subsequently, the absolute pupil diameters are used to determine the estimation error of the three selected pupil models.

### Prediction accuracy of chromatic light stimuli

**Figure 2:**
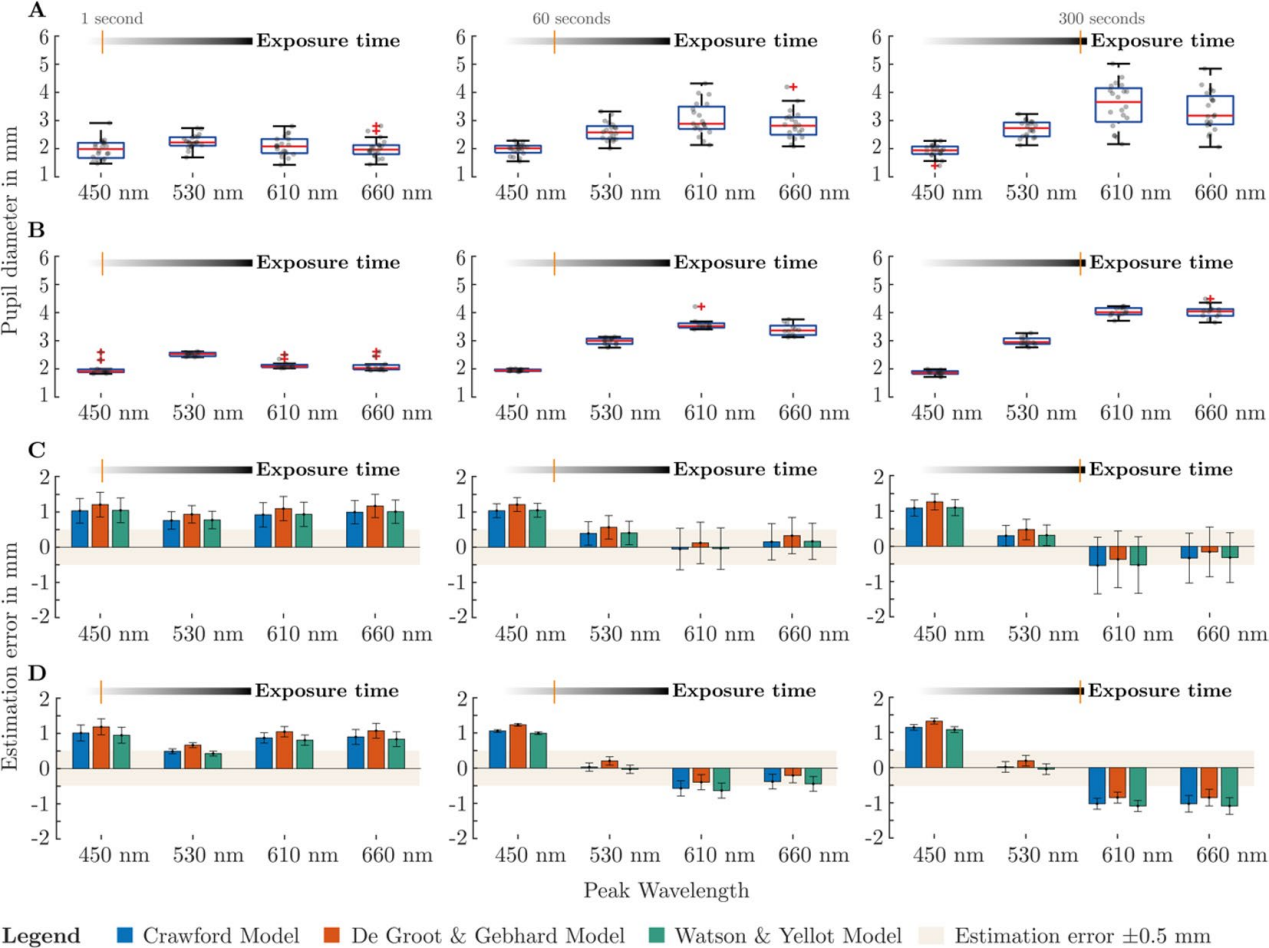
Measured pupil diameter and estimation error of pupil models from the inter- and intrapersonal experiment with chromatic stimuli. (**A**) Pupil diameter at exposure times of 1 s, 60 s and 300 s from the interpersonal experiment with 20 subjects (Age: 19–25, Mean age: 22.2 SD ± 1.77 years). (**B**) Pupil diameter from the intrapersonal experiment with one 33-year-old observer and 12 repetitions. (**C**) The mean and standard deviation of the differences between the calculated pupil diameter of the models and the measured pupil diameter from the interpersonal experiment with twenty subjects. The ribbon shows the estimation error ± 0.5 mm as an indicator. (**D**) Mean and standard deviation of the estimation error from the intrapersonal experiment.

The absolute pupil diameter from the interpersonal examination with chromatic stimuli showes that with increasing exposure time the influence of the wavelength becomes more notable (Fig. 2A,B). To check whether these diameter differences between used spectra are statistically significant, we performed a repeated measure ANOVA on the baseline-corrected pupil diameter (Supplementary materials Fig. S1A,B). Baseline correction was performed with the corresponding pupil diameter from the anchor spectrum, which we used to stimulate the observers, 300 s before the respective primary stimulus occurs (Supplementary materials Fig. S2A,B). According to graphical analysis with quantile–quantile plot and Shapiro–Wilk-Test, normal distribution of the pupil data in the interpersonal experiment with chromatic stimuli can be assumed. When conducting a statistical analysis on the interpersonal data from the chromatic experiment with one second exposure time (Fig. 2A left), Mauchly’s test indicated that the assumption of sphericity had been met with ${\chi }^{2}$(5) = 1.87, p = 0.86 > 0.05. Therefore, a correction of degree is not necessary. According to repeated measure ANOVA (rANOVA), the pupil diameter is significantly affected by the type of the given spectrum F (3, 57) = 12.24, p = 2.73 × 10^–6^ < 0.05 with a medium effect size ${\eta }^{2}\hspace{0.17em}$= 0.22. Pairwise comparison with Bonferroni correction reveals, that significant differences are between 450 and 530 nm (p = 2.49 × 10^–4^ < 0.05), but the baseline-corrected mean difference $\lvert \Delta {\stackrel{-}{\mu }}_{B} \lvert$ between 530 and 450 nm is quite small with 0.32 mm. Thus, with such a small error, a description of the pupil diameter with the luminance can be made at one second exposure time, since the pupil diameter has remained approximately constant across the used wavelengths.

At 60 s exposure time (Fig. 2A middle), Mauchly’s test showed that the assumption of sphericity had been met ${\chi }^{2}$(5) = 3.12, p = 0.68 > 0.05. According to rANOVA, there are significant differences between the used spectra F (3, 57) = 42.24, p = 1.67 × 10^–14^ < 0.05 with a large effect size ${\eta }^{2}\hspace{0.17em}$= 0.58. Pairwise t-test with Bonferroni correction shows significant differences between 450 and 530 nm (p = 1.68 × 10^–6^ < 0.05, $\lvert\Delta {\stackrel{-}{\mu }}_{B}\lvert\hspace{0.17em}$= 0.69 mm), 450–610 nm (p = 6.4 × 10^–8^ < 0.05, $\Delta {\stackrel{-}{\mu }}_{B}\hspace{0.17em}$= 1.11 mm) and 450–660 nm (p = 6.4 × 10^–8^ < 0.05, $\lvert\Delta {\stackrel{-}{\mu }}_{B}\lvert\hspace{0.17em}$= 1.11 mm). Therefore, the pupil response to 450 nm at 60 s exposure time is significantly smaller than with the other used wavelengths, meaning a generalized pupil model cannot depend on the luminance alone. A spectral weighting of short wavelengths, probably a dynamic ipRGC weighting, would be necessary here. At 300 s exposure time (Fig. 2A right), Mauchly’s test indicated that the assumption of sphericity had been violated ${\chi }^{2}$(5) = 18.79, p = 2.14 × 10^–3^ < 0.05. Therefore, the quite conservative Greenhouse–Geisser correction was applied. Repeated measure ANOVA shows that the pupil diameter is affected by the type of used spectra F (2.22, 42.2) = 50.81, p = 1.70 × 10^–12^ < 0.05 with a large effect size ${\eta }^{2}$= 0.61. Pairwise t-test with Bonferroni correction reveals significant differences between 450 and 530 nm (p = 1.58 × 10^–7^ < 0.05, $\lvert \Delta {\stackrel{-}{\mu }}_{B}\lvert\hspace{0.17em}$= 0.85 mm), 450–610 nm (p = 4.91 × 10^–8^ < 0.05, $\lvert \Delta {\stackrel{-}{\mu }}_{B}\lvert\hspace{0.17em}$= 1.66 mm), 450–660 nm (p = 4.03 × 10^–7^ < 0.05, $\lvert\Delta {\stackrel{-}{\mu }}_{B}\lvert\hspace{0.17em}$= 1.46 mm). While the pupil diameter at 450 nm remains nearly constant across exposure time, dilatation of the pupil can be observed at longer wavelengths, causing the increased average difference of the pupil diameter compared to the short wavelength. The statistical results from the intrapersonal experiment with one subject are reported in the Supplemental Material and agree with those from the interpersonal experiment. However, the individual subject showed higher averaged pupil differences between 450 nm and the longer wavelengths as the exposure time progressed. These results are within the scattering of the interpersonal experiment. In general, we found that some subjects showed a higher pupil dynamic between wavelengths, meaning a higher difference in pupil diameter between the used spectra. This is particularly evident in the fact that the scattering of pupil data increases with higher exposure time at longer wavelengths. In contrast, the scattering at the 450 nm stimulus remained nearly constant.

The pupil models expressed a nearly constant and increased error of 0.77–1.09 mm, depending on the used model, in the interpersonal experiment with one second exposure time. It is particularly interesting that the error remains almost constant, regardless of the used model and wavelength, as the statistical analysis of the baseline-corrected pupil diameter already revealed (Fig. 2C left). Thus, it is possible to determine the correct pupil diameter, by using an offset-correction of existing models. Especially in such a phasic time-range response of the pupil, L- and M-cone based models have the potential to predict diameter with an acceptable error, without having a large significant wavelength dependency. As the stimulus’ exposure time progresses, the forecast errors for the longer wavelengths decreases and are approximately within a tolerance range of ± 0.5 mm, independent of the used model (Fig. 2C middle, right). A higher estimation error is particularly visible at 450 nm, remaining even at longer exposure times between 1.02 and 1.26 mm. This effect becomes apparent when looking at the measured pupil diameter (Fig. 2A), in which the adaption state of a pupil’s control path is completed earlier and remains nearly constant. Pupil light response of other used wavelengths took longer exposure times to reach the steady-state equilibrium. Mapping this process inside a pupil model, a dynamic and time-dependent receptor weighting would particularly be helpful. The results from the intrapersonal experiment (Fig. 2D) are similar but showed a greater wavelength dependence at one second exposure time. Estimation error of the models for the wavelengths 450 nm, 630 nm and 660 nm lies between 0.83 mm and 1.01 mm. At 530 nm, there is a lower error of 0.42–0.66 mm, depending on the used model, since the mean pupil diameter is slightly larger at the other used wavelengths. The estimation error with a 450 nm stimulus is approximately constant across the exposure time with 0.94–1.31 mm. It is noticeable that in the intrapersonal examination, the estimation error increases from 60 to 300 s exposure time, which results in a higher estimation error at the end. The unfinished adaptation process has a larger significant impact on the estimation error in the intrapersonal examination (Fig. 2D middle, right).

Concerning the question of which model performs more robust, it can be concluded that there are only minimal differences between the models. The higher number of dependent parameters in the Watson and Yellot model did not lead to a significant improvement in pupil prediction. When comparing these models, the handier functions of Crawford and De Groot and Gebhard are a greater advantage than the additional parameter in the Watson and Yellot model. The parameter size of adaptation area may not show its advantages, because we are using a larger surface as it was used in the examinations behind models from Crawford and De Groot and Gebhard. From our investigations, we can conclude that having the parameter time dependency with a dynamic receptor weighting factor, is more crucial for a model. At least this statement applies to our experimental conditions. However, it can be assumed that these parameters will remain important regardless of the used luminance.

### Prediction accuracy with polychromatic light stimuli

**Figure 3:**
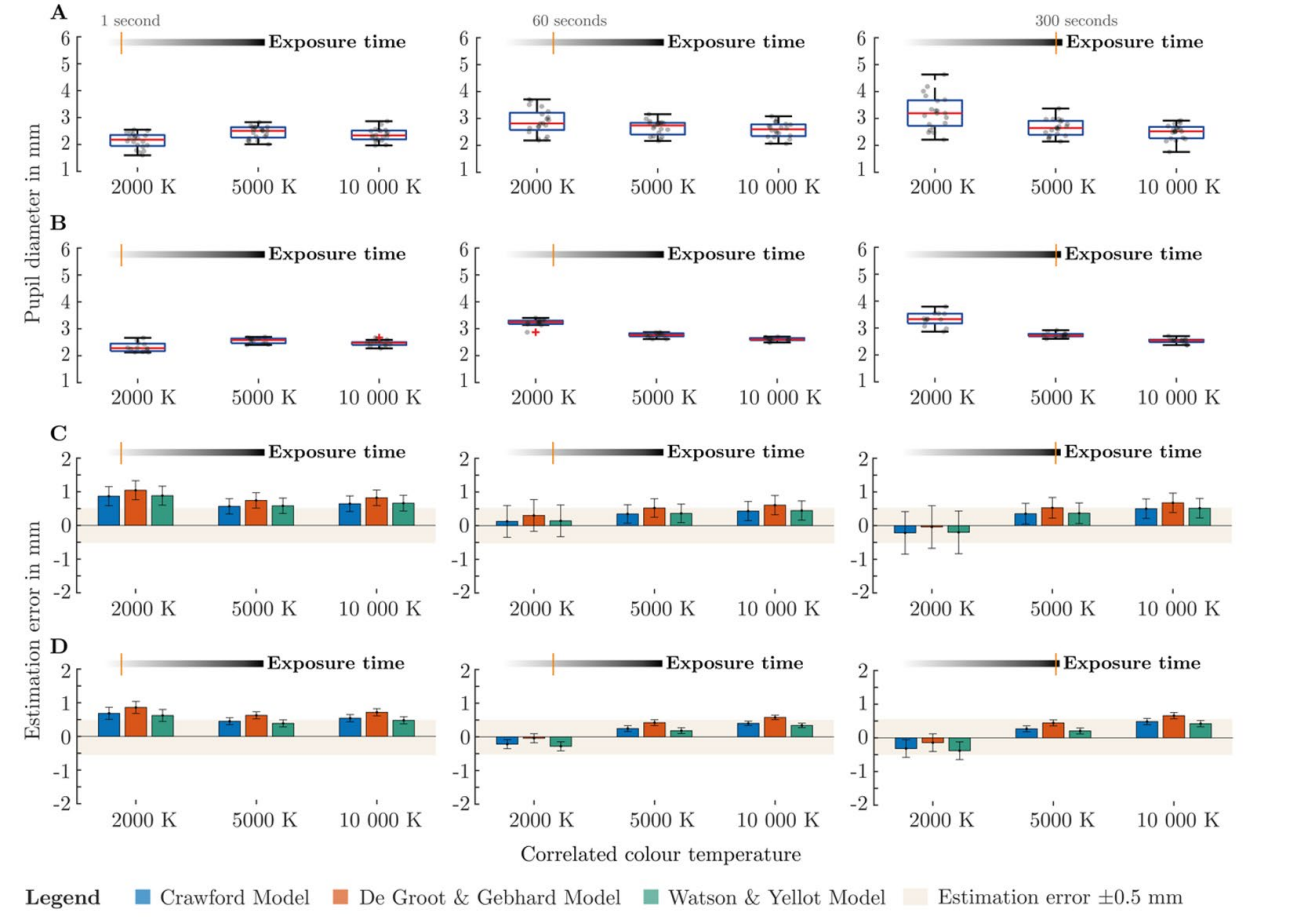
Measured pupil diameter and estimation error of used pupil models from the inter- and intrapersonal experiment with polychromatic stimuli. (**A**) Pupil diameter at exposure times of 1 s, 60 s and 300 s from the interpersonal experiment with 20 subjects (Age: 19–25, Mean age: 21.95 SD ± 1.73 years). (**B**) Pupil diameter from the intrapersonal experiment with one 33-year-old observer and 12 repetitions. (**C**) The mean and standard deviation of the differences between the calculated pupil diameter of the models and the measured pupil diameter from the interpersonal experiment with twenty subjects. The ribbon shows the estimation error ± 0.5 mm as an indicator. (**D**) Mean and standard deviation of the estimation error from the intrapersonal experiment.

Using chromatic spectra can be seen more like a special case to verify the limits of a pupil model. Polychromatic spectra along the Planckian curve correspond more to the types of light that is found indoors. With increasing exposure time, the pupil dilatation becomes larger for spectra with a lower ipRGC-signal. This effect, which was already evident in the chromatic experiment, is also visible here (Fig. 3A,B). However, because of the lower contrast of the melanopsin signal between 10 000 and 2000 K, the differences between the average pupil diameter are not so substantial as in the chromatic examination. As in the first study, the statistical analysis was carried out with the baseline corrected pupil diameter (Supplementary materials Fig. S1C,D). Baseline correction was performed with the corresponding pupil diameter from the anchor spectrum (Supplementary materials Fig. S2C,D). According to graphical analysis with quantile–quantile plot and the Shapiro–Wilk-Test, we can assume normal distribution of the pupil data in the interpersonal experiment with chromatic stimuli. Statistical analysis of the interpersonal experiment with polychromatic spectra at one second exposure time (Fig. 3A left), revealed that the assumption of sphericity has been met according to Mauchly’s test with ${\chi }^{2}$(2) = 0.77, p = 0.67 > 0.05. Repeated measure ANOVA showed that the pupil diameter is significantly affected by the type of spectrum F (2, 38) = 24.67, p = 1.36 × 10^–7^ < 0.05 with a large effect size ${\eta }^{2}\hspace{0.17em}$= 0.41. Pairwise t-test with Bonferroni correction revealed a significant difference between pupil diameter from 10 000 and 2000 K (p = 1.71 × 10^–3^ < 0.05, $\lvert\Delta {\stackrel{-}{\mu }}_{B}\lvert \hspace{0.17em}$= 0.19 mm), whereby the difference of the mean value between 10 000 and 2000 K is so small that a constant pupil diameter is approximately assumable across spectra. This assumption can be made for chromatic as well as polychromatic spectra with an exposure time of one second.

This assumption can be made for chromatic as well as polychromatic spectra with an exposure time of one second. At 60 s exposure time (Fig. 3A middle), Mauchly’s test indicated that the assumption of sphericity has been met ${\chi }^{2}$(2) = 0.35, p = 0.83 > 0.05. Repeated measure ANOVA showed that the pupil diameter is significantly affected by the used spectra F (2, 38) = 10.24, p = 2.77 × 10^–4^ < 0.05 with a medium effect size ${\eta }^{2}\hspace{0.17em}$= 0.23. Pairwise t-test with Bonferroni correction revealed significant differences of the pupil diameter between 10 000 and 2000 K (p = 1.55 × 10^–3^ < 0.05, $\lvert\Delta {\stackrel{-}{\mu }}_{B}\lvert\hspace{0.17em}$= 0.33 mm), but 10 000 to 5000 K is still not significantly different at 60 s exposure time (p = 0.43 > 0.05, $\lvert\Delta {\stackrel{-}{\mu }}_{B}\lvert\hspace{0.17em}$= 0.1 mm). Thus, in contrast to chromatic spectra, using polychromatic spectra leads to a nearly constant mean pupil diameter, even with longer exposure times. The difference of average between the baseline-corrected pupil diameters of 10 000–2000 K is still relatively small (0.33 mm). At 300 s exposure time (Fig. 3A-right), the assumption of sphericity has been violated ${\chi }^{2}$(2) = 8.79, p = 0.01 < 0.05. Therefore, we used the quite conservative Greenhouse–Geisser correction. Repeated measure ANOVA showed significant influence of the used spectra on the pupil diameter F (1.44, 27.4) = 20.95, p = 1.68 × 10^–5^ < 0.05 with a large effect size ${\eta }^{2}$=0.42. Pairwise t-test with Bonferroni correction revealed significant pupil diameter differences between 10 000 to 2000 K (p = 2.13 × 10^–4^ < 0.05, $\lvert\Delta {\stackrel{-}{\mu }}_{B}\lvert\hspace{0.17em}$= 0.74 mm), but still no significance between 10 000 K and 5 000 K (p = 0.12 > 0.05, $\lvert\Delta {\stackrel{-}{\mu }}_{B}\lvert\hspace{0.17em}$= 0.17 mm). When using the luminance as a parameter to predict the diameter at longer adaptation times, an error must be expected with polychromatic stimuli. However, the deviations of the pupil diameter by using polychromatic spectra are generally more acceptable than in the chromatic experiment. The statistical analysis from the intrapersonal examination comes to similar results, but with higher deviations of the pupil diameter between the spectra (see supplementary materials). This is due to the low scattering, which makes the spectral dependency of the pupil more apparent. The measured pupil diameters of the individual observer are within the dispersion of the interpersonal test, which shows that our sample is a good generalized representation of the pupil light response.

When benchmarking the pupil models, it appeared that the estimation performance with polychromatic spectra was better than in the chromatic experiment. Overall, the pupil prediction at 60 and 300 s (Fig. 3C middle, right) is pretty good and lies mostly inside the tolerance ribbon of ± 0.5 mm. At 300 s, the models show a deviation of 0.2–0.67 mm, depending on the spectrum and model (Fig. 3C right). The highest deviation at longer exposure times is caused by the De Groot and Gebhard Model at 10 000 K with 0.67 mm (300 s) and 0.60 mm (60 s). At one second exposure time (Fig. 3C left), the most substantial estimation error occurred (0.86–1.04 mm), when using 2000 K as stimuli. Surprisingly, with one second exposure time, the pupil diameter is better predicted at 10 000 K than 2000 K. The results from the intrapersonal examination are similar to those from the interpersonal examination, with a lower standard deviation of the estimated error (Fig. 3D). Overall, the models from Crawford and Watson and Yellot showed slightly less error in predicting the pupil diameter, comparing to De Groot and Gebhard. Integrating a time-dependent parameter inside a pupil model would have more potential than a dynamic melanopsin factor. At least this relation can be seen in polychromatic spectra. However, when analyzing the data, the results of polychromatic and chromatic stimuli should not be considered separately, as a unified pupil model should take into account all stimulus possibilities. Especially with the polychromatic spectra, it has been shown that the pupil control path's temporal adaptation behavior depends strongly on the used spectrum. The adaptation mechanism cannot be modeled by an ipRGC weighting alone since the pupil diameter tends to require a longer adaptation time for spectra with longer wavelength components. In general, the statement that L- and M-cone based pupil models perform significantly better in polychromatic spectra than in chromatic spectra is not true. At one second exposure time (interpersonal trial), the average estimation error from the model of Watson and Yellot for chromatic spectra was 0.94 SD ± $$0.12 $$mm, while it was 0.71 mm$$\hspace{0.17em}\pm \hspace{0.17em}0.15$$ for polychromatic spectra. Thus, the time component is an important factor and cannot be compensated by an ipRGC weighting alone. Such weightings should be in conjunction with a time-dependent component. However, it should be mentioned that we did not optimize the spectra for a possible maximal ipRGC-signal, meaning that the average pupil difference between 2000 and 10 000 K can be even higher. The potential gain of an ipRGC-signal depends on the used number of LED-channels in a luminaire, as a higher number of channels allows a higher degree of freedom in increasing the melanopsin signal while maintaining the correlated color temperature.

## Discussion

Since the discovery of the intrinsic photosensitive ganglion cells and their role in controlling the pupil light response, research has focused on the neurological opponent system in the inner retina to reveal the mechanism behind the constriction and dilatation pathway of the pupil. According to recent findings, the pupil has a wavelength sensitivity which does not correspond to the photopic luminous efficiency function V(λ) alone^26,27^. With an increased exposure time of a stimulus, the peak sensitivity shifts from approximately 510 to 470 nm^30,31^. In the photopic adapted eye, ipRGCs are mainly responsible for maintaining the sustained pupil constriction^95^. The phasic pupil light response depends on the achromatic channel consisting of L- and M-cones with an additional inhibitory contribution of S-^65–67^ and M-cones^68,69^. A transfer of these discovered findings to a practically appliable pupil model has been overlooked in pupil research so far. From 1926 to 2012, authors proposed eight empirical pupil models, which mainly depend on a V(λ) weighted quantity. The latest model from Watson and Yellot was focused on developing a unified model which incorporates the parameters: dependency of age, size of adaptation area and the number of exposed eyes. Our work aimed to show the impact of recent findings in pupil research on existing L- and M-cone based pupil models. The weaknesses of these models are particularly apparent in LED luminaires, where the spectrum can be systematically modified. We set our focus on the deviation of the predicted pupil diameter from the measured one, considering the time dependence and the spectrum of a stimulus. We also wanted to find the conditions under which the luminance could be used in a pupil model. The luminance has the advantage of getting a first estimation of the pupil size with no previous knowledge of a spectrum. Therefore, we conducted two experiments with chromatic (450 nm, 530 nm, 610 nm, 660 nm) and polychromatic (~ 10 000 K, ~ 5000 K, ~ 2000) LED spectra. All spectra were presented at constant luminance to find the conditions under which the pupil diameter is not affected by the spectra. Such a condition would make the luminance to an ideal candidate in predicting the pupil diameter correctly without knowing the spectrum behind a stimulus. We found that although there are statistically significant differences in both, chromatic and polychromatic spectra at one second exposure time, an approximately constant averaged pupil diameter can be assumed for different spectra. The most considerable averaged difference in baseline-corrected pupil diameter was between 530 and 450 nm with 0.32 mm and 10 000–2000 K with 0.19 mm. In this time range, it would be indeed possible to predict the pupil diameter with the luminance, resulting in minimal estimation errors. When evaluating the models from Crawford, De Groot and Gebhard and Watson and Yellot, we found that the largest miscalculation of the pupil diameter lies in this time range. The errors were almost constant across the used spectra, ranging from 0.77 to 1.03 mm for chromatic and 0.56–1.4 mm for polychromatic stimuli, depending on the wavelength and model. However, with an offset correction, it would be possible to predict the pupil diameter at one second exposure time correctly, using one of the classical pupil models. In the interpersonal study, the average prediction error of the Watson and Yellot model was 0.94 SD ± 0.12 mm for chromatic spectra and 0.71 SD ± 0.15 mm using polychromatic spectra. The offset value can be calculated from the two mean values and subtracted from the prediction of the Watson and Yellot model. The offset-corrected Watson and Yellot model for one second exposure time would thus have a smaller averaged prediction error of 0.12 SD ± 0.12 mm for the chromatic spectra and − 0.12 SD ± 0.15 mm for the polychromatic spectra. Such a simple offset correction can be used to calculate the initial pupil diameter as a condition for a model, which could predict the complete time response from such a starting point. For the other exposure times, a naive offset correction is no longer possible because the tonic pupil diameter has a time-dependent ipRGC weighting. With increasing exposure time, the pupil dilates increasingly with chromatic stimuli of the wavelengths 610 nm and 660 nm, while at 450 nm there is almost no change in pupil diameter. Surprisingly, the pupil models predict the diameter for the wavelengths 530 nm, 610 nm and 660 nm relatively well and are within the tolerance range of estimation error (± 0.5 mm). A higher deviation of 1.02–1.08 remains at 450 nm for longer exposure times. This illustrates the issue that pupil models do not consider the influence of the ipRGCs on the sustained diameter. The data showed that the pupil has not reached its steady-state at 60 s, so in a future model, a dynamic time-dependent ipRGCs weighting would have to be used to map this effect. Interestingly, the steady-state is reached much faster at 450 nm than at longer wavelengths. This effect is evident in intrapersonal experiments with chromatic stimuli. Some subjects show a stronger pupil dilatation under 660 nm than others, meaning that a novel pupil model would have greater estimation error in predicting the diameter for long wavelengths. This was shown by the fact that the scattering of pupil data is greater at long wavelengths than at short ones. There are only slight differences between the pupil models and a significantly improved prediction by an increase of the parameters “eye number”, “size of stimulus area” and “age” is not visible. The polychromatic examination showed that the low ipRGC contrast led to a reduced dependence of the pupil on the used color temperature. In general, the models seem to give good predictions, all within the tolerance range of ± 0.5 mm for longer exposure times such as 60 s and 300 s. Due to the increased errors at short exposure times of polychromatic spectra, the weakness of these models is mainly due to the non-existent time dependence. However, the next step is to develop a unified time-dependent pupil model for both chromatic and polychromatic spectra. Our investigation found that polychromatic spectra with short presentation times can have the same prediction errors as chromatic spectra. Thus, the initial hypothesis that the pupil diameter is generally better predicted with white spectra along the Planckian locus is not correct for all exposure times. When discussing a new pupil model, it must be noted that even complex pupil models that would include all pupil path effects must remain user friendly. If the application difficulty exceeds the advantage of a new model approach, then such a model will not be widely used. The spectrum of a light source is not always known, because the luminance can be determined much more quickly. In a recent work about circadian rhythmic, it has been shown that a spectrally dependent parameter such as the circadian stimulus can be modeled and approximated by colorimetric quantities^96^. Such correlations may also be possible in the field of pupil modeling. Pupil modeling with different motivations will likely meet in future publications. With the addition of luminance and colorimetric quantities, practically applicable models may allow an improved prediction for simple calculations. In contrast, developed neurophysiological receptor signal-based models will be used mainly for scientific purposes to simulate the effects of synaptic connections in the inner and outer retina. Thus, each approach has its own requirements. Besides the classic pupil models, Spitschan^80^ showed with data from Bouma^26^ that the Watson and Yellot model could benefit when melanopsin weighted radiant flux is used for the steady-state pupil diameter. However, no time component has been taken into account in this approach, as the Bouma data do not provide any information about the adaptation time. Our study has shown that the pupil's adaptation process cannot be described without a temporal parameter, especially when it comes to different distributed spectra compositions. A further model approach by Rao et al. used a melatonin action factor for ipRGC weighting to take the spectral influence for the tonic pupil diameter into account^97^. However, his model is based exclusively on phosphor-converted white LED light sources, which may reduce the generality of the spectral dependence. The Rao et al. model used the melatonin suppression sensitivity (circadian sensitivity function) $c\left( \lambda \right)$. The circadian sensitivity function $c\left( \lambda \right)$ can be derived with different approaches and is not standardized^52,53,98–100^. Apart from that, our data showed that a model that is based on polychromatic spectra does not offer a significant advantage when considering the higher effort in using such a model, compared to classical L- and M-cone approaches. When using polychromatic spectra, the most elevated errors occur in the phasic pupil diameter. However, such a time component has not been integrated. An additional melanopic component to extent current models or develop novel approaches can only reach an advantage when the temporal behavior is mapped with it.

Overall, our work has shown which errors can be expected when the pupil diameter is calculated from the luminance alone using classical models. We have kept the luminance constant and a conclusion about the receptor portions on the prediction error cannot be drawn from our results. Furthermore, we used in our work only one luminance level at 100 cd/m^2^. It would be interesting to check the accuracy of classical pupil models at the boundaries of high and low intensity ranges. These investigations are time-consuming due to the longer exposure time and can actually be directly linked to the development of a pupil model since pupil data under different spectra and luminance steps are the basic data requirement to build a model. From our point of view, the next step is to find out to what extent a maximized ipRGC-signal could influence the pupil diameter at constant chromaticity coordinates and luminance with a multichannel LED luminaire. The work of Tsujimura et al. showed that the ipRGC signal contributes by factor of three times more to the pupil constriction than the L- and M-cone signals^101^. Thus, it would be possible to optimize the interior illumination for the visual acuity by influencing the pupil diameter without changing the luminance. A spectral and time-dependent pupil model would be the key requirement for this.

## Supplementary information

Supplementary file1 (PDF 570 kb)

## Data Availability

The data that support the findings of this study are available from the corresponding author, upon reasonable request.
