# Supplementary material for: Prediction accuracy of L- and M-cone based human pupil light models
Source: Sci Rep. 2020 Jul 3;10:10988. doi: 10.1038/s41598-020-67593-3 (PMC7335057; doi:10.1038/s41598-020-67593-3)
Supplement: Supplementary file 1 — Supplementary file1 (PDF 570 kb) [file 41598_2020_67593_MOESM1_ESM.pdf]

Supplementary materials of the paper “Prediction accuracy of L- and M-cone based human pupil light models”. Authors: Babak Zandi, Julian Klabes and Tran Quoc Khanh

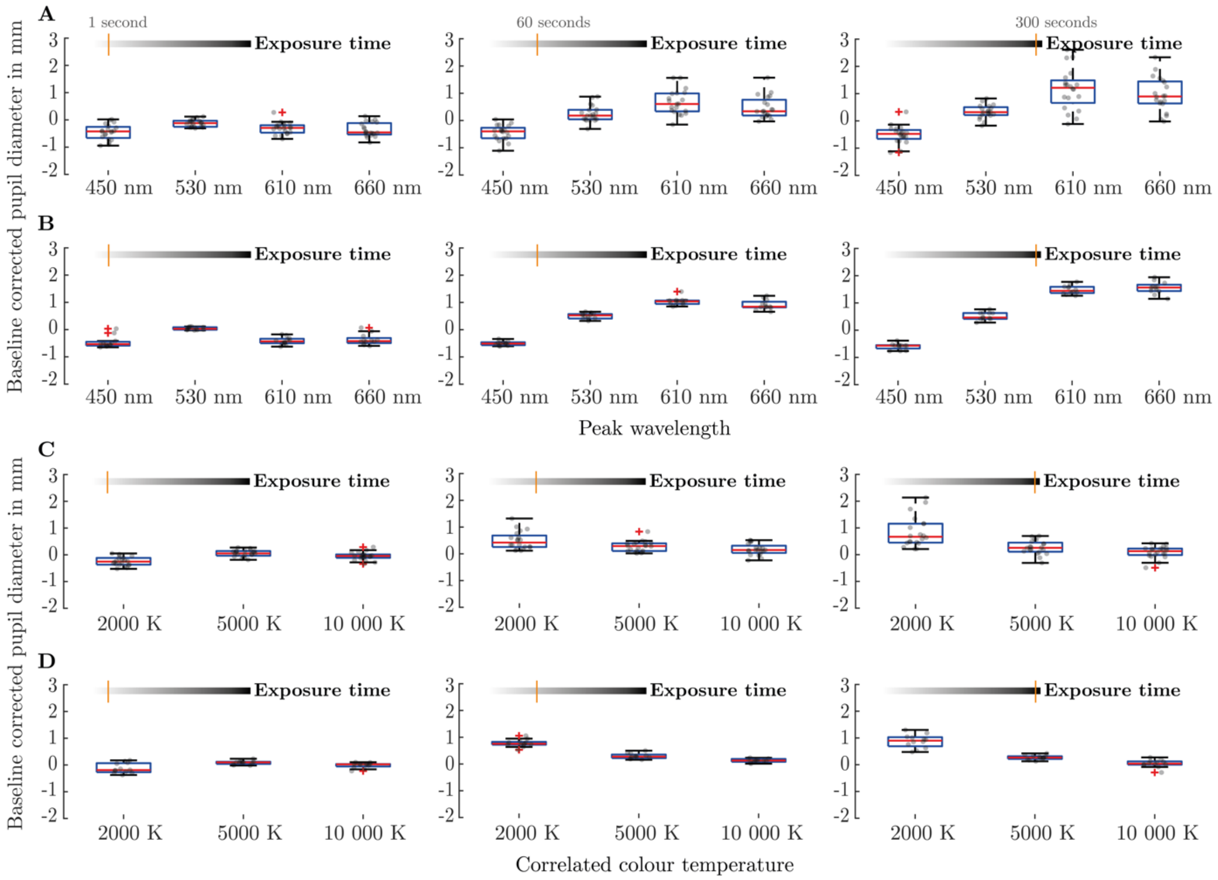

Figure S1 Baseline corrected pupil diameter of the chromatic and polychromatic experiment. **A:** Baseline corrected pupil diameter at exposure times of 1 second, 60 seconds and 300 seconds from the interpersonal experiment with 20 subjects (Age: 19-25, Mean age: 22.2 SD ±1.77 y). Baseline correction was performed with the pupil diameter from the anchor spectrum that came before the main stimulus with an exposure time of 300 seconds. The average value of the last second was used as a baseline pupil diameter. **B**: Pupil diameter from the intrapersonal experiment with one 33-year-old observer and 12 repetitions. **C:** Baseline corrected pupil diameter from the interpersonal experiment with 20 subjects (Age: 19-25, Mean age: 21.95 SD ±1.73 y). **D**: Pupil diameter from the intrapersonal experiment with one 33-year-old observer and 12 repetitions.

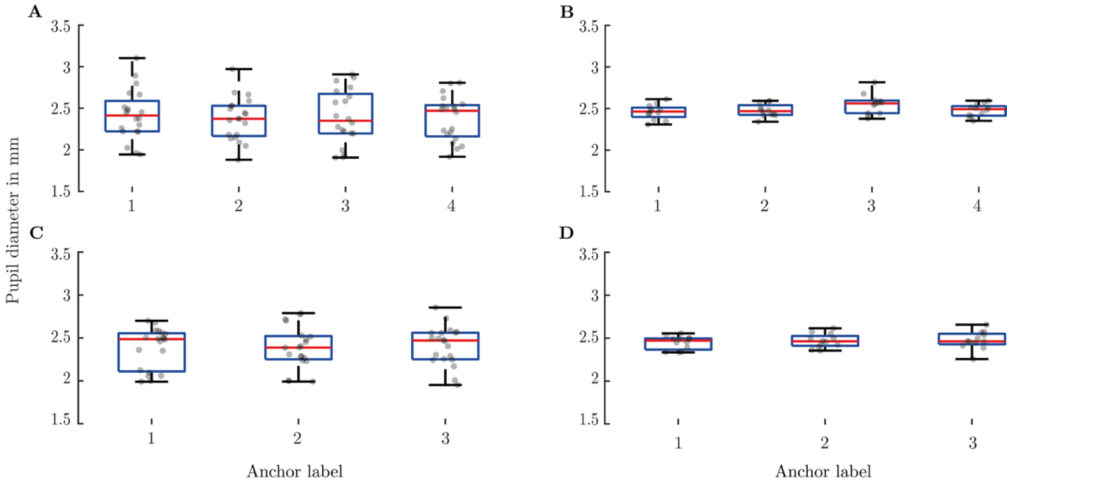

Figure S 2 Baseline pupil diameter of the anchor spectrum from the inter- and intrapersonal experiments with chromatic and polychromatic spectra. The averaged pupil diameter of the last second is shown. The exposure time was 300 seconds. The labels on the x-axis show the assignment of the pupil diameters that were measured in the anchor stimulus before the respective main stimulus. **A:** Baseline pupil diameter from the chromatic experiment. Anchor label 1 was before 450 nm, label 2 before 530 nm, label 3 before 610 nm and label 4 before 660 nm. **B:** Baseline pupil diameter from the intrapersonal chromatic experiment. The labels are the same as in B. **C:** Baseline pupil diameter from the polychromatic spectra. Anchor label 1 was before the 2000 K stimuli, label 2 before 5000 K and label 3 before 10 000 K. **D:** Baseline pupil diameter from the intrapersonal polychromatic experiment with the same label order labels as C.

Statistical analysis of the intrapersonal experiment with chromatic stimuli

According to graphical analysis with quantile-quantile plot and Shapiro-Wilk-Test, normal distribution of the pupil data in the intrapersonal experiment with chromatic stimuli can be assumed. When conducting a statistical analysis on the data of the interpersonal chromatic experiment with one second exposure time (Fig. S1 B left), Mauchly's test indicated that the assumption of sphericity had been violated with $\chi^2(5) = 11.26$, $p = 0.05 < .05$. Therefore, the quite conservative Greenhouse-Geisser correction is applied. According to repeated measure ANOVA, the pupil diameter is significantly affected by the type of spectrum $F(1.8, 19.86) = 49.48$, $p = 3.38 \cdot 10^{-8} < .05$ with a large effect size $\eta^2 = 0.62$. Pairwise t-test with Bonferroni correction showed a significant difference between 450 and 530 nm ($p = 4.29 \cdot 10^{-5} < .05$, $|\Delta\bar{\mu}_B| = 0.5$ mm). At 60 seconds exposure time (Fig. S1 B middle), Mauchly's test indicated that the assumption of sphericity had been met $\chi^2(5) = 10.5$, $p = 0.06 > .05$. According to rANOVA, there are significant differences between the used spectra $F(3, 33) = 298.87$, $p = 5.52 \cdot 10^{-24} < .05$ with a large effect size $\eta^2 = 0.96$. Pairwise t-test with Bonferroni correction show significant differences between 450 to 530 nm ($p = 1.74 \cdot 10^{-9} < .05$, $|\Delta\bar{\mu}_B| = 1.01$ mm), 450 to 610 nm ($p = 3.8 \cdot 10^{-11} < .05$, $|\Delta\bar{\mu}_B| = 1.53$ mm) and 450 to 660 nm ($p = 5.73 \cdot 10^{-10} < .05$, $|\Delta\bar{\mu}_B| = 1.42$ mm). At 300 seconds exposure time (Fig. S1 B right), Mauchly's test indicated that the assumption of sphericity had been met $\chi^2(5) = 10.94$, $p = 0.053 > .05$. According to rANOVA, there are significant differences between the used spectra $F(3, 33) = 511.1$, $p = 1.02 \cdot 10^{-27} < .05$ with a large effect size $\eta^2 = 0.97$. Pairwise t-test with Bonferroni correction show significant differences between 450 to 530 nm ($p = 3.88 \cdot 10^{-10} < .05$, $|\Delta\bar{\mu}_B| = 1.10$ mm), 450 to 610 nm ($p = 1.06 \cdot 10^{-13} < .05$, $|\Delta\bar{\mu}_B| = 2.07$ mm) and 450 to 660 nm ($p = 1.61 \cdot 10^{-10} < .05$, $|\Delta\bar{\mu}_B| = 2.15$ mm).

Statistical analysis of the intrapersonal experiment with polychromatic stimuli

According to graphical analysis with quantile-quantile plot and Shapiro-Wilk-Test, normal distribution of the pupil data in the intrapersonal experiment with polychromatic stimuli can be assumed. At one second exposure time (Fig. S1 **D-left**), Mauchly's test indicated that the assumption of sphericity had been met with $\chi^2(5) = 4.59$, $p = 0.1 > .05$. According to repeated measure ANOVA, the pupil diameter is significantly affected by the type of spectrum $F(2, 22) = 61.24$, $p = 1.02 \cdot 10^{-9} < .05$ with a large effect size $\eta^2 = 0.72$. Pairwise t-test with Bonferroni correction showed no significant difference between 10 000 K and 2000 K ($p = 0.77 > .05$, $|\Delta\bar{\mu}_B| = 0.1$ mm). However, a significant difference occurs between 10 000 K and 5000 K ($p = 1.59 \cdot 10^{-6} < .05$, $|\Delta\bar{\mu}_B| = 0.1$ mm). At 60 second exposure time (Fig. S1 D middle**)**, Mauchly's test indicated that the assumption of sphericity had been met with $\chi^2(5) = 0.13$, $p = 0.93 > .05$. According to repeated measure ANOVA the pupil is affected by the type of spectrum $F(2, 22) = 521.26$, $p = 2.92 \cdot 10^{-19} < .05$ with a large effect size $\eta^2 = 0.97$. Pairwise t-test with Bonferroni correction showed significant differences between 10 000 K to 2000 K ($p = 1.9 \cdot 10^{-11} < .05$, $|\Delta\bar{\mu}_B| = 0.64$ mm) and 10 000 K to 5000 K ($p = 5.62 \cdot 10^{-7} < .05$, $|\Delta\bar{\mu}_B| = 0.16$ mm). At 300 seconds exposure time (Fig. S1 **D-right)**, Mauchly's test indicated that the assumption of sphericity had been met with $\chi^2(5) = 2.57$, $p = 0.27 > .05$. According to repeated measure ANOVA the pupil is affected by the type of spectrum $F(2, 22) = 901.69$, $p = 7.79 \cdot 10^{-22} < .05$ with a large effect size $\eta^2 = 0.97$. Pairwise t-test with Bonferroni correction showed significant differences between 10 000 K to 2000 K ($p = 5.32 \cdot 10^{-14} < .05$, $|\Delta\bar{\mu}_B| = 0.83$ mm) and 10 000 K to 5000 K ($p = 1.31 \cdot 10^{-8} < .05$, $|\Delta\bar{\mu}_B| = 0.22$ mm).

Table S 1 Measured absolute spectra in W/m^{2}sr of the main and anchor stimuli used in the experiment. Spectra were measured with a Konica Minolta CS2000 spectroradiometer. Wavelength step size was reduced to 5 nm for a compact visualization.

Wavelength	Anchor 5500 K	2000 K	5000 K	10 000 K	450 nm	530 nm	610 nm	660 nm
380	2.69E-06	2.47E-06	2.95E-06	3.79E-06	6.62E-05	7.51E-07	7.18E-06	7.51E-05
385	1.92E-06	0.00E+00	0.00E+00	0.00E+00	1.65E-04	2.23E-06	1.34E-06	5.41E-05
390	0.00E+00	0.00E+00	0.00E+00	0.00E+00	6.78E-05	5.28E-07	0.00E+00	2.13E-05
395	0.00E+00	0.00E+00	0.00E+00	5.38E-07	1.49E-05	1.42E-06	0.00E+00	1.24E-06
400	0.00E+00	0.00E+00	4.04E-06	1.43E-05	2.46E-05	0.00E+00	1.37E-06	3.52E-05
405	7.45E-06	9.20E-07	1.84E-05	8.26E-05	3.17E-04	3.67E-07	2.52E-07	2.66E-05
410	2.92E-05	3.62E-06	7.17E-05	3.17E-04	1.20E-03	1.19E-06	0.00E+00	1.09E-05
415	1.00E-04	6.69E-06	2.11E-04	9.70E-04	3.85E-03	0.00E+00	6.96E-06	1.04E-05
420	2.62E-04	1.39E-05	4.60E-04	2.11E-03	9.95E-03	0.00E+00	0.00E+00	3.48E-05
425	5.88E-04	2.10E-05	5.94E-04	2.50E-03	2.23E-02	7.14E-07	0.00E+00	1.05E-05
430	1.14E-03	2.76E-05	5.41E-04	1.75E-03	4.20E-02	2.71E-06	0.00E+00	6.40E-06
435	2.01E-03	4.24E-05	6.88E-04	1.67E-03	7.27E-02	2.36E-06	0.00E+00	2.76E-05
440	3.55E-03	7.22E-05	1.12E-03	2.35E-03	1.18E-01	1.04E-06	0.00E+00	1.01E-05
445	5.47E-03	1.19E-04	1.73E-03	3.39E-03	1.54E-01	1.24E-06	1.57E-07	3.10E-06
450	5.13E-03	1.52E-04	1.85E-03	3.35E-03	1.30E-01	2.96E-06	0.00E+00	9.04E-06
455	3.09E-03	1.50E-04	1.46E-03	2.27E-03	7.58E-02	2.74E-06	0.00E+00	1.85E-07
460	2.28E-03	1.20E-04	1.22E-03	1.80E-03	4.43E-02	3.83E-06	0.00E+00	2.11E-06
465	1.79E-03	1.00E-04	1.24E-03	1.84E-03	2.66E-02	7.34E-06	4.40E-07	0.00E+00
470	1.23E-03	9.49E-05	1.42E-03	2.13E-03	1.44E-02	1.33E-05	0.00E+00	1.06E-05
475	1.07E-03	9.50E-05	1.54E-03	2.38E-03	8.24E-03	2.76E-05	0.00E+00	0.00E+00
480	1.12E-03	1.11E-04	1.40E-03	2.12E-03	4.99E-03	5.81E-05	1.81E-06	0.00E+00
485	1.22E-03	1.63E-04	1.16E-03	1.61E-03	2.92E-03	1.18E-04	0.00E+00	1.44E-05
490	1.47E-03	2.58E-04	1.08E-03	1.35E-03	1.73E-03	2.34E-04	0.00E+00	1.36E-05
495	1.79E-03	4.16E-04	1.20E-03	1.38E-03	1.07E-03	4.55E-04	0.00E+00	3.75E-06
500	2.10E-03	6.40E-04	1.44E-03	1.58E-03	7.11E-04	8.53E-04	0.00E+00	1.32E-05
505	2.36E-03	8.95E-04	1.71E-03	1.86E-03	4.77E-04	1.54E-03	0.00E+00	1.27E-05
510	2.57E-03	1.12E-03	1.88E-03	2.08E-03	3.38E-04	2.54E-03	1.36E-06	3.24E-06
515	2.72E-03	1.27E-03	1.89E-03	2.17E-03	2.63E-04	3.75E-03	3.71E-06	0.00E+00
520	2.83E-03	1.36E-03	1.83E-03	2.17E-03	2.21E-04	4.80E-03	2.65E-06	1.26E-06
525	2.90E-03	1.37E-03	1.73E-03	2.08E-03	1.81E-04	5.18E-03	1.20E-06	1.13E-06
530	2.96E-03	1.26E-03	1.60E-03	1.87E-03	1.62E-04	4.72E-03	0.00E+00	0.00E+00
535	3.02E-03	1.09E-03	1.44E-03	1.58E-03	1.46E-04	3.75E-03	0.00E+00	5.74E-07
540	3.06E-03	9.40E-04	1.31E-03	1.32E-03	1.24E-04	2.82E-03	2.51E-06	1.46E-05
545	3.10E-03	8.48E-04	1.22E-03	1.15E-03	1.37E-04	2.10E-03	1.20E-06	0.00E+00
550	3.12E-03	8.06E-04	1.16E-03	1.04E-03	1.23E-04	1.55E-03	2.59E-06	4.10E-06
555	3.12E-03	8.12E-04	1.12E-03	9.74E-04	1.19E-04	1.11E-03	8.26E-06	1.89E-06
560	3.12E-03	8.47E-04	1.10E-03	9.47E-04	1.08E-04	7.69E-04	1.54E-05	7.89E-06
565	3.10E-03	9.15E-04	1.10E-03	9.57E-04	1.15E-04	5.34E-04	2.78E-05	1.13E-06
570	3.06E-03	9.93E-04	1.10E-03	9.83E-04	1.16E-04	3.74E-04	5.42E-05	1.73E-05
575	3.01E-03	1.08E-03	1.10E-03	1.01E-03	9.38E-05	2.65E-04	1.02E-04	1.23E-05
580	2.95E-03	1.16E-03	1.11E-03	1.04E-03	1.10E-04	1.85E-04	1.94E-04	1.08E-05
585	2.90E-03	1.23E-03	1.13E-03	1.06E-03	1.02E-04	1.28E-04	3.64E-04	1.96E-05
590	2.83E-03	1.32E-03	1.14E-03	1.08E-03	1.07E-04	8.98E-05	6.72E-04	2.02E-05
595	2.77E-03	1.44E-03	1.18E-03	1.09E-03	1.05E-04	6.35E-05	1.22E-03	5.38E-05
600	2.69E-03	1.63E-03	1.23E-03	1.10E-03	8.92E-05	4.54E-05	2.17E-03	1.45E-04
605	2.60E-03	1.97E-03	1.35E-03	1.13E-03	7.59E-05	3.23E-05	3.82E-03	3.68E-04
610	2.49E-03	2.58E-03	1.55E-03	1.19E-03	8.50E-05	2.51E-05	6.67E-03	7.61E-04
615	2.37E-03	3.60E-03	1.89E-03	1.32E-03	9.97E-05	1.85E-05	1.11E-02	1.37E-03
620	2.24E-03	5.05E-03	2.37E-03	1.52E-03	7.55E-05	1.39E-05	1.67E-02	2.38E-03
625	2.10E-03	5.96E-03	2.61E-03	1.75E-03	8.57E-05	1.12E-05	1.81E-02	4.01E-03
630	1.95E-03	4.89E-03	2.13E-03	1.77E-03	7.83E-05	8.15E-06	9.26E-03	6.70E-03
635	1.81E-03	3.03E-03	1.52E-03	1.49E-03	8.13E-05	7.52E-06	3.20E-03	1.09E-02
640	1.66E-03	1.95E-03	1.20E-03	1.36E-03	8.33E-05	6.49E-06	1.11E-03	1.68E-02
645	1.51E-03	1.81E-03	1.17E-03	1.56E-03	8.61E-05	5.34E-06	3.95E-04	2.64E-02
650	1.37E-03	2.18E-03	1.32E-03	2.04E-03	7.81E-05	4.48E-06	1.61E-04	4.14E-02
655	1.23E-03	2.79E-03	1.56E-03	2.68E-03	7.16E-05	5.03E-06	7.69E-05	5.89E-02
660	1.10E-03	2.53E-03	1.41E-03	2.44E-03	9.32E-05	3.91E-06	3.60E-05	6.20E-02
665	9.75E-04	1.22E-03	8.26E-04	1.19E-03	7.54E-05	4.56E-06	2.27E-05	3.21E-02
670	8.59E-04	6.65E-04	5.56E-04	6.54E-04	7.85E-05	1.90E-06	1.54E-05	1.19E-02
675	7.55E-04	4.61E-04	4.37E-04	4.61E-04	5.75E-05	3.81E-06	3.50E-06	4.63E-03
680	6.58E-04	3.85E-04	3.71E-04	3.98E-04	5.30E-05	2.68E-06	1.01E-05	1.91E-03
685	5.75E-04	3.71E-04	3.27E-04	3.93E-04	9.03E-05	4.49E-06	6.64E-07	8.47E-04
690	5.03E-04	3.85E-04	2.95E-04	4.28E-04	7.69E-05	3.99E-06	3.66E-06	4.22E-04
695	4.37E-04	4.40E-04	2.72E-04	5.05E-04	7.92E-05	2.66E-06	0.00E+00	2.16E-04
700	3.75E-04	5.38E-04	2.59E-04	6.41E-04	6.32E-05	1.68E-07	2.35E-06	1.12E-04
705	3.27E-04	7.00E-04	2.55E-04	8.54E-04	4.58E-05	8.08E-07	5.69E-06	9.29E-05
710	2.77E-04	9.13E-04	2.62E-04	1.14E-03	1.00E-04	1.01E-06	9.61E-08	5.11E-05
715	2.35E-04	1.18E-03	2.78E-04	1.47E-03	9.45E-05	3.77E-06	9.68E-06	3.32E-05
720	2.05E-04	1.45E-03	2.97E-04	1.82E-03	7.46E-05	4.12E-06	8.51E-06	4.54E-05
725	1.72E-04	1.78E-03	3.27E-04	2.26E-03	1.31E-04	1.14E-06	1.04E-05	5.59E-05
730	1.51E-04	2.17E-03	3.64E-04	2.77E-03	1.23E-04	6.14E-06	7.71E-06	3.36E-05
735	1.27E-04	2.32E-03	3.72E-04	2.96E-03	7.77E-05	3.33E-06	0.00E+00	2.62E-05
740	1.11E-04	1.68E-03	2.82E-04	2.14E-03	1.20E-04	4.36E-06	8.44E-06	4.46E-05
745	9.63E-05	8.17E-04	1.62E-04	1.02E-03	1.24E-04	5.50E-06	1.27E-05	3.51E-05
750	8.42E-05	3.72E-04	1.00E-04	4.49E-04	1.72E-04	5.18E-06	1.45E-05	7.30E-08
755	7.10E-05	1.85E-04	7.20E-05	2.20E-04	2.25E-04	1.33E-05	1.54E-05	1.07E-04
760	6.29E-05	1.18E-04	6.06E-05	1.20E-04	1.97E-04	1.95E-05	1.92E-05	6.25E-05
765	4.61E-05	8.51E-05	5.61E-05	7.98E-05	1.96E-04	3.03E-05	3.75E-05	1.25E-05
770	4.27E-05	7.72E-05	6.27E-05	6.76E-05	4.07E-04	4.17E-05	3.43E-05	6.62E-05
775	3.14E-05	8.11E-05	7.02E-05	7.11E-05	2.51E-04	4.46E-05	5.21E-05	1.65E-04
780	3.78E-05	9.46E-05	8.75E-05	6.89E-05	7.03E-04	7.09E-05	9.28E-05	1.47E-04
